# Harnessing the Microbiome to Prevent Fungal Infections: Lessons from Amphibians

**DOI:** 10.1371/journal.ppat.1005796

**Published:** 2016-09-08

**Authors:** Jenifer B. Walke, Lisa K. Belden

**Affiliations:** Department of Biological Sciences, Virginia Tech, Blacksburg, Virginia, United States of America; Geisel School of Medicine at Dartmouth, UNITED STATES

All multicellular organisms are host to microbial symbionts that constitute the microbiome and can have significant impacts on the host, including altering development, behavior, and health [1]. In turn, aspects of the host and their environment can influence the microbiome [2]. Here, we briefly summarize current knowledge of the amphibian skin microbiome and its role in heath and disease. Given the increase in fungal diseases that now threaten amphibians and other wildlife—including bees, bats, snakes, and corals, as well as a variety of economically important crops [3]—we hope that lessons learned from amphibian host–microbe interactions can also ultimately be applied in other systems (Fig 1).

**Fig 1:**
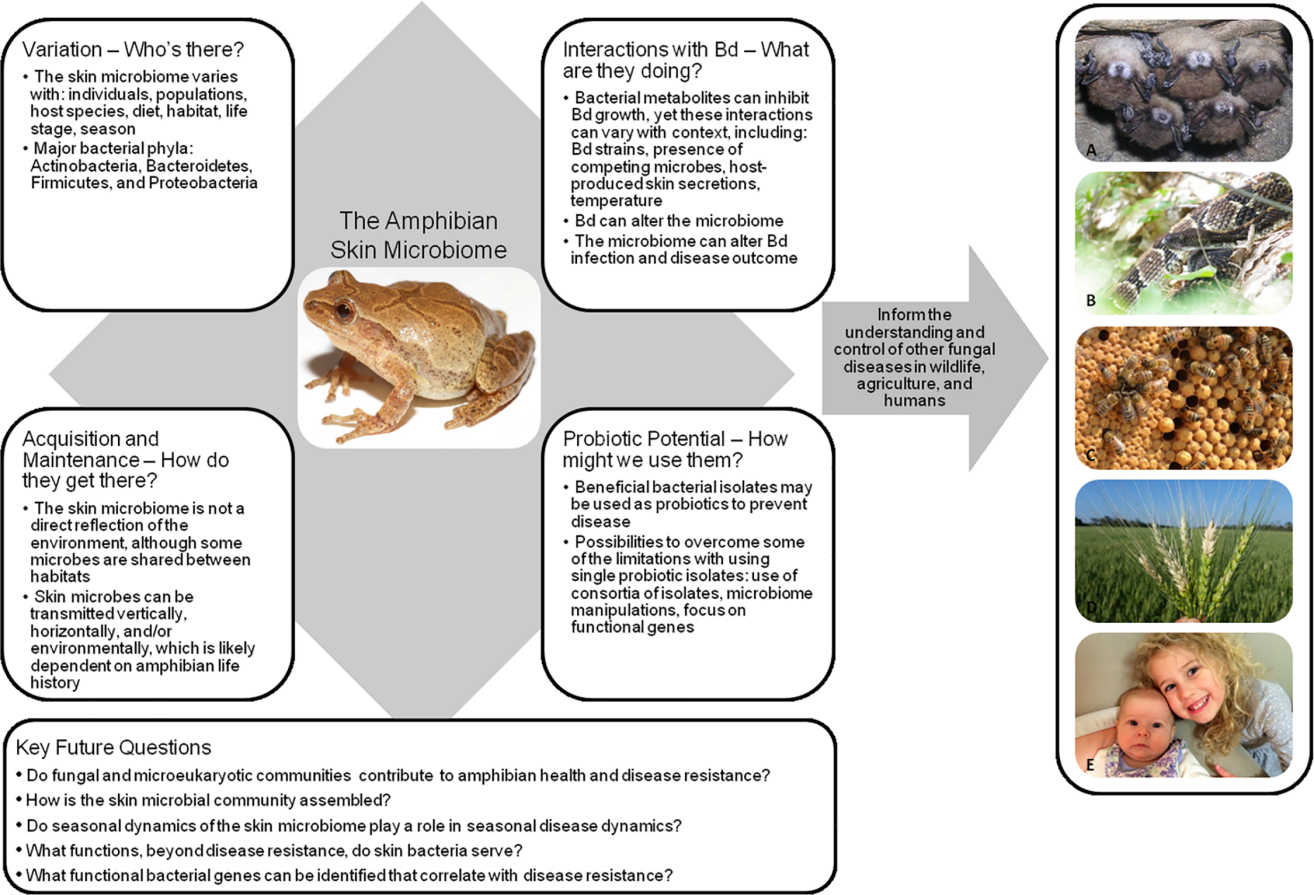
Overview of the amphibian skin microbiome and its interactions with the fungal pathogen, *Batrachochytrium dendrobatidis* (Bd, Chytridiomycota). Lessons learned in the amphibian–microbiome–fungal pathogen system may be applied to other organisms affected by fungal diseases, including (A) bats (white-nose syndrome caused by *Pseudogymnoascus destructans*, Ascomycota), (B) snakes (snake fungal disease caused by *Ophidiomyces ophiodiicola*, Ascomycota), (C) bees (*Nosema* sp., Microsporidia), (D) wheat (e.g., wheat blast caused by *Magnaporthe oryzae*, Ascomycota), and (E) humans (e.g., *Trichophyton rubrum*, Ascomycota). Photo credits: (frog) Brian Gratwicke, (A) United States Geological Survey National Wildlife Health Center, (B) Anne G. Stengle, (C) Richard Fell, (D) Guillermo Isidoro Barea Vargas, (E) Jenifer Walke.

## Why Study the Amphibian Skin Microbiome?

Most knowledge about the amphibian skin microbiome has been derived from researchers trying to mitigate the effects of the potentially lethal skin disease chytridiomycosis, which is caused by the fungal pathogen *Batrachochytrium dendrobatidis* (Bd) [4]. Bd attacks the skin and has caused numerous amphibian population declines and extinctions around the world [4]. In fact, chytridiomycosis has caused the greatest documented loss of biodiversity because of a disease [3]. Skin bacteria likely play a role in host resistance and immune function, along with host life history, genetics, behavior, and antimicrobial peptides. If we can harness the power of these microbes, we might be able to prevent disease [5].

## Variation in the Skin Microbiome: Who’s There?

Skin microbiome structure varies across amphibian species, populations, and individuals (e.g., [6,7]). Factors such as diet [8] and habitat [9] can shape the overall community composition, species richness, and relative abundance of skin bacteria. Many amphibians undergo a dramatic change in physiology and habitat use during metamorphosis, and shifts in the microbiome have been observed across host life stages (e.g., [7,10,11]). Immune function and skin characteristics also change during metamorphosis, which may influence microbial community assembly. Vulnerability to Bd is often highest among recently metamorphosed amphibians, and shifts in the microbiome may play an important role in that. The amphibian skin microbiome might also change seasonally in both temperate zones, where temperatures vary dramatically through the year, and in tropical regions with strong wet and dry seasons [10]. This, in turn, could contribute to the timing of Bd outbreaks. Despite the wide range of variation in microbial community structure and the methods used to characterize these communities, the following bacterial phyla are consistently dominant on amphibian skin, although with varying relative abundances: Actinobacteria, Bacteroidetes, Firmicutes, and Proteobacteria [12]. Thus far, most of the focus on the amphibian skin microbiome has been on bacterial communities, but fungal communities and other eukaryotes could also play an important role in disease resistance [11].

## Interactions with Bd: What Are They Doing?

The skin, including the microbiome, serves as a first line of defense against invading pathogens, such as Bd. In vitro assays, in vivo laboratory experiments, and field surveys suggest that amphibians’ skin microbiome plays an important role in their innate immune system. The protective effect of the microbiome is likely due to bacterial metabolites inhibiting Bd zoospore colonization or development (e.g., indole-3-carboxaldehyde and violacein produced by *Janthinobacterium lividum* [13]), which may be a byproduct of competition among skin microbes [14]. In vitro assays are a useful method for evaluating the anti-Bd function of bacterial isolates [15], but many bacterial isolates do not consistently inhibit growth of a broad range of Bd strains [16]. Also, most screening for anti-Bd bacterial isolates is done with pure cultures, yet when bacteria are grown in mixed communities, as they exist on amphibian skin, different secondary metabolites can be produced [17]. For example, the emergent anti-Bd metabolite tryptophol was produced when *Bacillus* sp. and *Chitinophaga arvensicola* were grown together but not when either was grown in isolation [17]. Furthermore, the ability of these bacterial isolates to inhibit Bd can be impacted by environmental conditions, including the presence of host-produced skin secretions, competition among microbes, pathogen presence, and temperature [18–21]. To account for some of this context-dependency, Woodhams et al. [19] developed a holistic, noninvasive assay to assess the anti-Bd function of the mucosome, which includes interactions among host-produced immune factors, the skin microbiome, and microbe-produced secondary metabolites. Even examining metabolite production in more complex bacterial communities might increase our ability to understand these interactions.

Experiments have demonstrated that an augmented protective microbiota can reduce morbidity and mortality in some amphibians exposed to Bd (e.g., [22]) and that the cutaneous microbial community can alter disease outcomes [23,24]. Bd infection can also alter the skin microbiome [23,25]. However, not all bioaugmentation experiments have been successful at protecting amphibians from Bd (e.g., [26]). In addition to experimental work, in field surveys, amphibian populations coexisting with Bd had a higher proportion of individuals with anti-Bd skin bacteria than populations experiencing declines (e.g., [27]), and Bd infection can influence—or be influenced by—the skin microbiome [10,25]. Several studies have also now correlated amphibian species’ susceptibility to Bd with the skin microbiome (e.g., [28]).

The skin microbiome may serve a defensive function against pathogens other than just Bd, including, for instance, cutaneous *Amphibiocystidium* parasites [29], fungal embryo pathogens [30], and the newly discovered fungal species *Batrachochytrium salamandrivorans* (Bsal), which infects salamanders and is also an emerging threat to global amphibian biodiversity. In addition, some of these microbes might serve completely different functions, beyond disease resistance, such as maintenance of the mucosal layer, toxin production, or vitamin synthesis. All of these functions could have important consequences for host fitness and likely vary with changes in environmental conditions.

## Acquisition and Maintenance: How Do They Get There?

Symbiotic bacteria can be acquired in a number of ways. First, microbes can be transmitted vertically from parent to offspring, though in amphibians this route of transmission is likely limited to species exhibiting parental care. For example, the microbiomes of four-toed salamanders and their guarded embryos were more similar to each other than either were to the microbes in the nest environment [30]. Amphibians may also pick up their skin microbes from the environment in each new generation or following disturbances, such as skin sloughing. While amphibians share some microbes with their environment, the amphibian skin microbiome is not simply a reflection of the environmental microbial community, and host factors appear to select for environmental microbes colonizing the skin (e.g., [6,7,31]). Bacteria can be transferred experimentally from soil to salamanders [32], and the available environmental microbial source pool is important for maintenance of the skin microbiome [31]. Lastly, skin microbes can be transmitted horizontally (i.e., host to host), both directly through contact among tadpole hosts and indirectly from host to environment to another host [33]. The amphibian gut, which also harbors diverse microbial communities [34], may serve as a reservoir for skin disease–fighting microbes. The amphibian skin microbiome is likely to be acquired, and then maintained, by a mixture of transmission modes that vary depending on amphibian life history.

## Probiotic Potential: How Might We Use Them?

Several tools currently exist for amphibian conservation of Bd-susceptible species, including captive breeding programs and chemical treatments. However, one applied, sustainable solution to the problem of conserving Bd-susceptible species in Bd endemic regions could lie in probiotic therapy. Using beneficial probiotic isolates, consortia of isolates, or microbiome manipulations to enhance protection of amphibians against infectious diseases, such as chytridiomycosis, has great potential. However, there are challenges associated with this approach, including the limited ability of single bacterial isolates to inhibit different strains of Bd [16], to protect different amphibian species (e.g., [22,26]), and to consistently provide host protection under a variety of contexts and environments [19,20]. Focusing on functional genes that produce anti-Bd metabolites to identify suites of candidate probiotics might be more successful than focusing on specific bacterial taxa. Furthermore, microbiome engineering [35] to generate a protective microbiome structure and/or function may be a useful strategy, as the use of complete microbiome transplants in humans can effectively limit disease [36].

The use of omics technologies has been powerful in advancing our understanding of amphibian host–microbiome–pathogen interactions and informing conservation efforts [5], but it is also clear that the study of bacterial isolates and their traits (e.g., anti-Bd capabilities) is critical. It is encouraging that many of the dominant or relatively abundant skin bacteria are culturable [37], thus allowing for the evaluation of functional traits of actual isolates. An open-access database has been established that consists of the 16S rRNA gene sequences and anti-Bd capabilities of cultured amphibian skin bacteria from a wide variety of amphibian species and geographical locations [38].

Researchers from a variety of disciplines—including ecology, microbiology, immunology, biochemistry, and amphibian biology—have worked collaboratively to battle chytridiomycosis. This approach of interdisciplinary research teams can serve as a model to advance discovery in the face of emerging pathogens. Many of the lessons revealed through this research can be applied in other organisms affected by fungal diseases, from wildlife to agricultural crops and even humans (Fig 1). Fungal diseases are increasing in incidence and are a major threat to biodiversity [3], yet they are notoriously difficult to treat. A probiotic approach, which naturally sustains itself, may be a powerful fungal infection–fighting tool to mitigate biodiversity loss.
